# The Use of Pulmonary Arterial Pressure (PAP) for Improved Beef Cattle Management

**DOI:** 10.3390/ani14162430

**Published:** 2024-08-22

**Authors:** Kaylen Stearns, Hannah DelCurto-Wyffels, Sam Wyffels, Megan Van Emon, Tim DelCurto

**Affiliations:** Department of Animal Science, Montana State University, Bozeman, MT 59717, USA; hannah.delcurto@montana.edu (H.D.-W.); sam.wyffels@montana.edu (S.W.); megan.vanemon@montana.edu (M.V.E.); timothy.delcurto@montana.edu (T.D.)

**Keywords:** beef cattle health, performance, pulmonary arterial pressure

## Abstract

**Simple Summary:**

An estimated USD 60 million is lost annually due to High Altitude Disease in the cow–calf industry, and there continues to be a growing concern about heart–lung function in feedlot cattle located at moderate elevations. As the cattle industry puts more emphasis on and focuses on heart–lung function, it is valuable to provide a review of past research and information gaps regarding pulmonary arterial pressure (PAP) and its use in the beef cattle industry.

**Abstract:**

Pulmonary arterial pressure (PAP) determines cattle’s susceptibility to High Altitude Disease (HAD), also known as Brisket Disease, High Mountain Disease, and right-sided heart failure (RHF). This non-infectious disease causes pulmonary hypertension due to hypoxia. PAP measures the resistance of blood flow through the lungs. It is estimated that 1.5 million head of cattle are raised in high-altitude environments (above 1500 m), and HAD accounts for 3–5% of calf death loss yearly. In addition, there have been increasing concerns about feedlot cattle succumbing to RHF at moderate elevations. This review focuses on the historical background, explanation of PAP measurement and scores, genetic implications, and the relationship between PAP and economically relevant traits. Specifically, traits such as gestation length, birth weight, weaning weight, and yearling weight may impact PAP scores. In addition, environmental effects and other factors impacting PAP score variations are discussed. Information gaps and research needs are addressed to determine where missing information could improve the understanding of PAP while also benefiting beef cattle producers in high-elevation production systems.

## 1. Introduction

**Figure 1:**
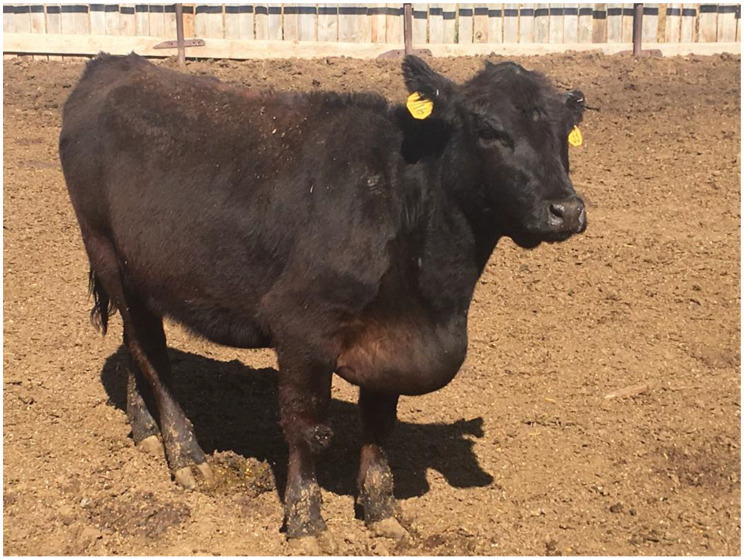
This heifer has High Altitude Disease. The noticeable edema begins at her head and continues down through her brisket—Photo Courtesy of Dr. Tim Holt, Colorado State University.

High Altitude Disease (HAD) is a non-transmissible, non-infectious disease that impacts beef cattle production at elevations above 1500 m due to low oxygen saturation in the atmosphere [1]. High Altitude Disease is also known as Brisket Disease, right-sided heart failure (RHF), and High Mountain Disease (HMD) [2]. The stress from the lack of oxygen can cause the animal to develop HAD [1]. High Altitude Disease causes hypoxic pulmonary vasoconstriction, and eventually, the animal will be sent into cardiac failure due to reduced heart–lung function [3]. The progression of this disease begins with alveolar hypoxia, then pulmonary vasoconstriction, which leads to pulmonary remodeling and eventually turns into pulmonary hypertension [1]. Eventually, hypertrophy of the right ventricle will occur, which causes dilation of the right ventricle, and this leads to right-sided heart failure [1]. Clinical signs of HAD include brisket edema, lethargy, jugular vein distention, diarrhea, poor appetite, and death (Figure 1) [4]. There is no treatment or cure for HAD; the only recommendation that exists is to move cattle to lower elevations [4]. While RHF is most common among cattle, left-sided heart failure can also occur. Left-sided heart failure presents clinical signs of respiratory stress, such as dyspnea and cough, due to the overload of the pulmonary circulation system [5]. However, the cardiovascular system is a closed circuit, so no matter which side of the heart fails first, eventually, it becomes bilateral heart failure [6]. Pulmonary arterial pressure (PAP) scores were developed to determine an animal’s susceptibility to HAD [2]. Pulmonary arterial pressure scores measure the resistance of blood flow through the lungs [7]. Beef cattle producers in high-elevation production systems have used PAP scores for several decades as a guide to make management decisions and determine an animal’s survivability in high-elevation environments [7,8,9].

## 2. History 

Cattle exhibiting clinical signs of what is now known as HAD were first reported in 1914 by Glover and Newsom [4] in Colorado. In 1913, Glover and Newsom were chosen by cattle ranchers in Colorado to explore what ranchers called Dropsy, or HMD [10]. These cattle resided in areas of high elevation (2743–3048 m) in Colorado [4]. The cattle displayed the same clinical signs of edema, poor appetite, dull appearance, lethargy, and death that are used to describe HAD currently [4]. 

Following two years of research, Glover and Newsom reached several conclusions about this physiological disease that are still held today [10]. The first conclusion was that altitude was the main cause of HAD [4]. Secondly, cattle who were transported from low elevations to high elevations were more susceptible, and these cattle should be gradually introduced to higher altitudes [4]. From a physiological standpoint, Glover and Newsom concluded that the hearts of cattle who were diagnosed with HAD were overworked [4]. Thus, Glover and Newsom [4] recommended that cattle experiencing these symptoms be moved to lower elevations. The same recommendation still exists for cattle exhibiting similar symptoms today [3]. Glover and Newsom [4] recommended using native bulls who were suited for the high elevations as herd sires to improve the “hardiness of the herd.” From these observations, Glover and Newsom (1914) made the assumption that an animal’s susceptibility to HAD was heritable [4].

## 3. Economic Impact

In 2012, it was estimated that 1.5 million head of cattle were raised in high-altitude environments [11]. High Altitude Disease accounts for 3–5% of calf death loss in high-elevation production systems yearly [2]. Given these figures, it is reported that HAD costs the cattle industry approximately USD 60 million each year, with an estimated loss of 75,000 head of cattle [11]. Research has shown that by evaluating PAP scores when selecting and retaining breeding stock, producers can help mitigate the risk of cattle developing HAD by selecting animals that are better suited for production systems at high elevations [7]. 

## 4. Increased Susceptibility of HAD in Bovine

Pulmonary vascular shunting occurs as a response in all animals to hypoxic conditions [2] and is described as persistent exposure of the pulmonary vasculature to increased pressure, which will result in severe pulmonary vascular disease [12]. Pulmonary vascular shunting acts to move pulmonary blood flow away from poorly oxygenated tissues in the lung to tissues with a higher oxygen content [13]. Comparing pulmonary vascular shunting across species revealed that cattle experienced the greatest shunting across the tested species of llamas, dogs, and cats [12].

The bovine pulmonary system is relatively small compared to its body weight and has a smaller lobulated anatomical pattern, which makes it more likely to suffer from severe pulmonary hypertension and loss of function [14]. The increase in pulmonary vascular shunting, combined with the anatomical features of the bovine lung, makes cattle much more susceptible to pulmonary hypertension caused by hypoxia [2,13]. Pulmonary vascular shunting will eventually lead to the remodeling of pulmonary arteries caused by hypertrophy and muscularization [13,15]. The increased resistance will cause a loss of function within the peripheral pulmonary arteries [13]. These changes within the pulmonary system cause hypertrophy and dilation of the right ventricle, which is followed by RHF [2,15].

## 5. Measuring Pulmonary Arterial Pressure

A pulmonary arterial pressure (PAP) score measures the resistance of blood flow through the lungs [7]. Similar to human blood pressure, PAP is measured in millimeters of mercury (mmHg) [2]. Scores will range from 30 mmHg to >50 mmHg, with lower scores being more desirable (30–40 mmHg) [2]. For accurate measurements, PAP scores should be taken at elevations of at least 1500 m and after cattle have spent at least three weeks residing at 1500 m to induce hypoxic conditions [2,16]. The accuracy of the test can be improved by residing at elevations greater than 1500 m for longer than 3 weeks [2]. Cattle should be greater than 12 months of age when tested to reduce variation, as it has been shown PAP scores will increase as an animal matures [2,17,18]. Therefore, the scores of animals who are tested at younger than 12 months of age are an inaccurate representation of the animal’s true PAP score [2]. 

Pulmonary arterial pressure measurements are performed through a chute-side, right-sided heart catheterization procedure by inserting a flexible catheter into the jugular vein [19]. The animal should be properly restrained within a squeeze chute, and the head should also be restrained [2]. It is important to note that the chute should not be squeezed too tight to ensure accurate results [2]. The pressure within the jugular vein is first measured [2]. For normal animals at 1500 m and above, the pressure within the jugular vein should be within 6–12 mmHg [2]. The catheter is passed through the right atrium and into the right ventricle [2]. Another measurement is taken with the right ventricle (RV), known as the RV measurement [2]. In healthy animals at 1500 m and above, the RV measurement should range from 18–30 mmHg [2]. Finally, the catheter reaches the pulmonary artery, where the PAP measurement is taken [2]. The mean PAP (mPAP) score is then measured from the pressure transducer connected to the end of the catheter in the pulmonary artery by measuring and taking the average of both the diastolic and systolic pressures [19].

## 6. Interpreting PAP Scores

Based on PAP scores and the elevation of the operation, some animals may be better suited for higher-elevation production systems compared to others. The mean PAP score can be used to determine the animal’s risk for developing HAD (Table 1). Holt and Callan [2] recommend that animals who have a PAP score of less than 41 mmHg at greater than 12 months of age can be retained as breeding stock [2]. Animals with a PAP score of greater than 49 mmHg at any age should not be retained in high-elevation production systems [2]. Cattle with PAP scores between 41 mmHg and 49 mmHg should be closely observed and used with caution in high-elevation operations [2]. Cattle at risk of developing HAD residing in high elevations will have PAP scores between 48 and 213 mmHg [2].

## 7. Factors Impacting PAP Score Variations

**Figure 2:**
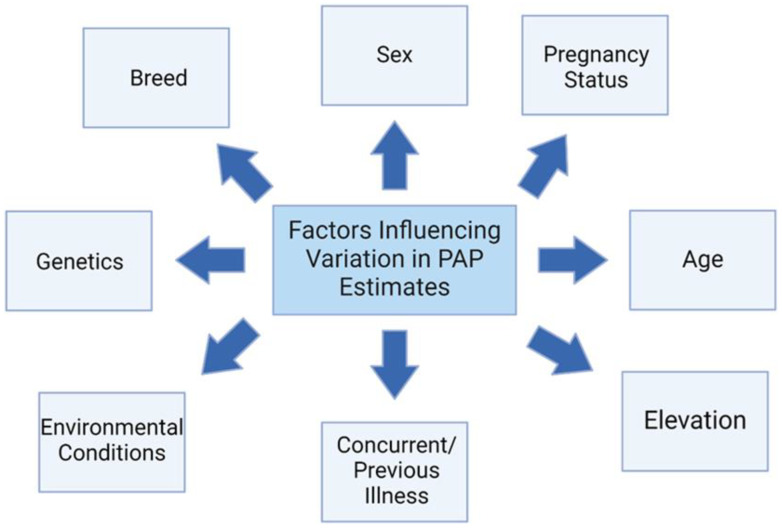
Factors that might influence beef cattle Pulmonary Arterial Pressure (PAP) estimates (adapted from Holt and Callan, 2007 [2]).

Several factors can influence PAP scores (Figure 2). When selecting and retaining breeding stock, Holt and Callan recommend considering several factors. While it has been shown that all breeds have cattle that vary greatly in PAP scores and all have animals with high PAP scores (>50 mmHg), some genetic lines within breeds perform better in high-elevation environments and have more desirable PAP scores [2]. A case study at the San Juan Basin Research Center 4 Corners Bull Test evaluated the factors influencing PAP across ten different breeds [20]. It was shown that breed (*p* < 0.001) was an important factor for considering variations in PAP scores [20]. Simmental bulls were shown to have the highest mPAP scores (53.1 ± 2.1) [20]. The authors attribute this result to the uniqueness of the Simmental bulls consigned to the bull test [20]. In addition, the authors also found this result surprising due to the fact that Simmental cattle originate from Switzerland, and it would be expected that this breed would be adapted to high-elevation environments [21]. Hereford Bulls and Polled Hereford Bulls had the lowest mPAP scores at 42.3 ± 0.4 and 42.3 ± 0.8, respectively [18]. However, another study conducted in 2020 showed that a cross between Angus and Red Angus cattle had the highest mPAP [22]. A comparison study between Angus and Salers cattle showed there are specific single nucleotide genomic polymorphisms (SNP) responsible for the genetic diversity that increases resilience to HAD [23]. Angus cattle in this study were sorted into a low PAP group (<41 mmHg) and a high PAP group (>41 mmHg), and even between the two groups of Angus cattle, it was revealed that there were differences in genetic frequencies [23]. Evidence suggests that an animal’s susceptibility to HAD is inherited [2].

A study by Moore et al. [24] showed that pregnant females who are susceptible to HAD had an increase in both PAP and blood flow resistance compared to pregnant females who are resistant to HAD. Females that were shown to be resistant to HAD had no change in their PAP scores [24]. Females with an already high PAP score will have an increased score when pregnant, which will put a greater strain on heart and lung function [24]. Animals experiencing a concurrent or past respiratory illness, parasites, or lung abscess may test with a higher PAP score, as these conditions can induce hypoxia and increase the risk of pulmonary hypertension [2].

When performing PAP tests, environmental factors can influence the outcomes. As mentioned previously, PAP scores should be taken at an elevation of 1500 m or higher to ensure accurate results [2,16]. Those who are measuring the PAP scores of cattle that are expected to be used as breeding stock in high-elevation production systems should pay special attention to the elevation at which the PAP scores are taken [2,16]. Cold environmental temperatures below 0 degrees Celsius can cause pulmonary hypertension and increase vascular resistance in cattle, which increases PAP scores [25]. 

Certain feedstuffs, such as ionophores and a certain alkaloid found in locoweed, can increase PAP scores. Cattle that are fed ionophores are shown to have greater PAP scores [2]. A small pilot study conducted at a feedyard in Colorado above 1500 m showed that 44% of calves had a PAP score of greater than 50 mmHg, and 71% had a PAP score of greater than 45 mmHg after being fed ionophores for 6 weeks per label recommendation [2]. After the removal of the ionophores from the diet, PAP scores decreased by a mean of 6 mmHg [2].

Swainsonine, an alkaloid found in locoweed, specifically species in the Astragalus and Oxytropis genus, has been shown to induce HAD following consumption in high-elevation environments [26]. Swainsonine was fed in the milk to a group of young Holstein bull calves at an elevation of 3090 m and compared to another group of bull calves that did not receive swainsonine in the milk [26]. All five calves that were fed swainsonine displayed clinical signs of HAD, including edema in the brisket and right ventricular hypertrophy [26]. Only one calf out of five in the control group displayed signs of HAD [26]. Holt and Callan [2] have reasoned that the increased instances of HAD are due to the cardiotoxic effects of swainsonine combined with hypoxia-induced pulmonary hypertension.

## 8. Concerns at Moderate Elevations

Recently, there has been an increase in RHF at moderate elevations considered to be below 1500 m [18]. From 2000 to 2012, the cases of RHF in feedyards across the United States and Canada have nearly doubled, making RHF the second leading cause of death in feedyards behind pneumonia [18]. The increase in RHF deaths comes as carcass weights in the United States have reached an all-time high [27]. Grain-fed cattle are shown to have the highest PAP scores [28]. Pulmonary arterial pressure scores of fattening feedlot cattle ranged from 42 to 143 mmHg [28]. Comparing the PAP scores of feedlot cattle to other classes of cattle showed that the mean PAP score of Angus feedlot cattle was 8.3 mmHg greater than yearling Angus bulls and 13 mmHg higher than grazing Angus heifers [28]. 

A case study conducted by Moxley and coworkers [29] discussed the reasons for the increase in RHF deaths in a feedlot located in western Nebraska. Cattle with pulmonary vascular lesions supported the idea that RHF is caused by hypoxia-induced pulmonary hypertension [29]. Furthermore, these cattle were kept at moderate elevations, which led researchers to believe that they were genetically predisposed to pulmonary hypertension because of hypoxia [29]. There was also an increase in intimal hyperplasia in pulmonary elastic arteries, which is consistent with HAD [19,29].

## 9. Alternative Methods of Measuring PAP

Measuring PAP scores can be seen as a possibly dangerous, invasive, and expensive procedure [19]. Due to the risks associated with the procedure, seedstock producers have been slow to adopt the practice [19]. Ahola and coworkers [19] conducted a study exploring alternative measurements for PAP scores using hemogram values, pulse oximetry, and a portable clinical analyzer. Hemogram values were shown to be the most reliable and accurate [19]. A blood sample was taken following the removal of the catheter after the PAP score measurement [19]. The morphology of red blood cells was evaluated [19]. Changes in red blood cell morphology and red blood cell distribution width can indicate increased erythropoiesis and vasoconstriction of the pulmonary vessels [30]. Past evidence has shown that cattle will modify their red blood cell characteristics to compensate for hypoxia [31]. In addition, previous research has shown cattle with increased PAP scores will have premature ventricular contractions, increased hemoglobin levels, red blood cell mass, and erythropoietin, a hormone responsible for signaling the need for more red blood cells [32]. Hemoglobin levels and red blood cell distribution proved to show the most promising results when evaluating blood samples compared to PAP scores [19]. More research should be evaluated to establish a definite correlation between red blood cell characteristics and PAP. 

The use of pulse oximetry was also evaluated and was a poor predictor of PAP scores [19]. Previous research conducted by Cueva [31] suggested that as hemoglobin levels were altered, it may also cause a change in blood oxygen saturation (SpO_2_). SpO_2_ values were collected at two locations on the cattle, and neither proved to be an accurate predictor of PAP. However, the researchers believe that the inability to refrain cattle from movement and the black hide color of the cattle may have impacted the accuracy of the pulse oximeter [19]. 

The portable clinical analyzer was also found to be a poor predictor of PAP scores [19]. Blood samples were taken from the cattle and immediately evaluated by the portable clinical analyzer [19]. Several blood samples were incorrectly evaluated due to immediate clotting, and no results were gathered from those samples [19]. The portable clinical analyzer evaluated 11 blood components, and no correlation was detected between blood components and PAP scores [19].

## 10. Inheritance of PAP 

Several studies have estimated the heritability of PAP in beef cattle and range from 0.20 to 0.46, respectively [7,8,9,33]. Variations have been reportedly caused by differences in age, sex, and breed type [33]. Bulls have a reported heritability for PAP of 0.31, and heifers reported a heritability of 0.20 [9]. In spring-born registered Angus cattle, PAP was found to be moderately heritable at 0.34 [7]. Environmental influences have also been shown to affect the heritability levels of certain traits due to changes in the phenotype of the animal [34].

Neary [35] conducted a genome-wide association study (GWAS) on PAP phenotype and genotype. The results showed that PAP is a polygenic trait, meaning that PAP is influenced by an estimated one thousand genes [35]. These genes were associated with a variety of pathways, including immune function, tissue remodeling, and metabolism [35]. Since PAP is a polygenic trait, an SNP-based predictor is required to predict performance, accurately calculate estimated progeny differences (EPD), and make genetic advancements [28].

## 11. PAP Scores as an EPD

In 2019, the American Angus Association (AAA), Angus Genetics Inc. (AGI), and Colorado State University (CSU) released an EPD for PAP scores for producers to follow and make informed genetic decisions [36]. International Genetic Solutions (IGS) and CSU also partnered together to create a multi-breed PAP EPD using Simmental, Red Angus, and commercial cattle [37]. The PAP EPD is used to determine an animal’s “survivability” in high-elevation environments [28] and is expressed in mmHg [36,37]. A lower score is more favorable and should indicate that a sire should produce offspring with a lower PAP score [36,37]. IGS reports that in 2020, the PAP EPD ranged in sires from −7.4 to 24.1 with a reported accuracy of 0.20 [37]. It is reported that 90% of cattle will have a PAP EPD ranging from −4 to +4 [28].

Both the AAA and IGS stress the point that a PAP EPD does not replace a PAP test for an animal’s survivability at high elevations due to the environmental factors that can impact an animal’s heart–lung function, such as illness, body condition, and other factors that can impact high-elevation beef cattle production [37]. The PAP EPD should be used to select parents of the next generation while mitigating risks for producers at high elevations [36]. Additionally, producers located at high elevations can select sires for artificial insemination (AI) and have background knowledge on how the expected offspring should handle the added stress of a high-elevation production setting [37].

## 12. Relationship between PAP Scores and Economically Relevant Traits

Seedstock producers use PAP scores as a marketing tool to sell seedstock animals to those producers who have cattle operations in high elevations and prevent RHF. As RHF continues to be a persistent issue in both moderate and high-elevation environments and the cattle industry pushes to better understand cattle heart health, it is necessary to determine how PAP estimates can impact traits that producers rely on for profit. Several studies have been conducted attempting to understand the relationship between PAP and economically relevant traits. To date, these studies have yielded conflicting results.

### 12.1. PAP Scores and Gestation Length

Selection for low birth weight sires and/or calving ease sires has been shown to directly decrease gestation length [38]. In contrast, as gestation length increases, it is estimated that birth weight increases by 0.25 kg/day [38]. The lungs are not used in utero and are some of the last tissues to develop [39]. Lung maturity at birth is directly correlated to fetal maturity and surfactant production [40], which supports the claim that shortened gestation lengths can directly impact lung function. Surfactant production helps promote a healthy pulmonary system by reducing surface tension and preventing the collapse of alveoli [41]. An immature pulmonary system can put a calf at greater risk for respiratory stress disease [42]. Furthermore, research has suggested that respiratory stress disease may put an animal at an increased risk of developing pulmonary hypertension [28].

A study by Foxworthy and coworkers [43] investigated the possible influence of a 286-day gestation length on yearling PAP scores. Foxworthy et al. [43] hypothesized that since lung development occurs in utero, gestation lengths may be a cause for variation in yearling PAP scores. Gestation lengths were measured from the point of artificial insemination to the calving date [43]. The relationship between gestation lengths and yearling PAP scores was then evaluated using the Pearson correlation coefficient and a simple linear regression model [44]. The study showed that a 286-day gestation length was a weak, negative predictor (−0.005) of yearling PAP scores and an animal’s susceptibility to HAD [43].

### 12.2. PAP Scores and Birth Weight

According to the AAA, the birth weight of the calf should be measured within 24 h of calving and should be measured in lbs./kgs. Putting an increased selection pressure on low birth weights to prevent dystocia has decreased birth weights and gestation lengths [38]. Similar to the gestation length discussion above, the health of the pulmonary system seems to be directly correlated to the size of the calf at birth [44]. Lighter birth-weight calves have been shown to have a compromised respiratory system, while heavier birth-weight calves are shown to be healthier and more resilient to stressors put on their respiratory system [44]. However, it should be noted that dystocia in high-birth-weight calves can compromise the animal’s viability and survivability due to a host of reasons, such as hypoxia, injury, inflammation, and others [45]. Dystocia has the ability to impact an animal’s health and performance throughout its lifespan [45].

Two conflicting studies have been reported defining a relationship between PAP scores and birth weights. Crawford et al. [33] used cattle that were owned by the Colorado State University—Beef Improvement Center (CSU-BIC) located near Encampment, Wyoming, for the research project, which aggressively culls for PAP scores above 42 mmHg. Cattle resided at an average elevation ranging from 2149 to 2410 m. Pulmonary arterial pressure scores were collected at an average of 356 days of age [33]. Data records from 1993 to 2014 were analyzed for this study. Crawford et al. reported a weak, positive genetic correlation (0.15 ± 0.09) between PAP scores and birth weights [33]. Crawford et al. [33] also suggested that selecting for lower PAP scores may decrease birth weights.

Similarly, Shirley et al. [7] used a database of PAP scores and body weights from a ranch in Colorado that resides at an average elevation of 1980 m. Pulmonary arterial pressure measurements were taken during weaning, and the average age was approximately 277 days [7]. Records were used from 1984 to 2003, with approximately 115 animals being PAP tested every year [7]. Shirley et al. [7] reported that PAP scores and birth weights were moderately and positively genetically correlated (0.49 ± 0.12) [7]. Unlike the study discussed above [33], it was concluded that selection for performance and growth at low altitudes may increase PAP scores and susceptibility to HAD if moved to high elevations [7]. Additionally, those who select for performance in high-elevation production systems should consider PAP scores when selecting for economically relevant traits [7,33].

### 12.3. PAP Scores and Weaning Weight

Previous work has also evaluated the relationship between PAP scores and weaning weights [7,33]. Crawford et al. [33] observed a weak to modest relationship (0.22 ± 0.08) between PAP scores and weaning weights [33]. Particularly for weaning weight, it was suggested that the selection for higher weaning weights could contradict the selection for lower PAP scores [33]. Shirley et al. [7] observed a moderate, positive genotypic correlation (0.50 ± 0.18) between PAP scores and weaning weights. Phenotypic correlations between PAP and weights were determined to be positive but low (0.02 ± 0.03) [7]. Both studies argued for caution when putting selection pressure on weaning weight for cattle residing in high-elevation environments and production systems [7,33].

### 12.4. PAP Scores and Yearling Weight

Crawford et al. [33] evaluated the relationship between PAP scores and yearling weights while also looking into the relationship between PAP scores and postweaning gain. There was found to be a weak genetic correlation (0.12 ± 0.08) between PAP scores and yearling weights, as well as postweaning gain [33]. An earlier study conducted by Schimmel [46] used records from multiple different breeds using a sire model analysis and showed there was a strong negative relationship (−0.75 ± 0.65) between PAP scores and yearling weights. 

Pauling et al. [47] also researched the relationship between PAP scores and yearling weights. A preexisting data set, including growth, carcass ultrasound records, and PAP scores from replacement heifers and bulls born between 1992 and 2015, was obtained from the AAA [47]. Cattle were separated into contemporary groups, and a 6-trait analysis was performed, resulting in a low genetic correlation (<0.20) between PAP scores and yearling weights [47].

### 12.5. PAP Scores and Carcass Traits

Pauling et al. [47] also evaluated the relationship between PAP scores and carcass traits using a previous data set made available by the AAA. It was hypothesized that increased selection pressure for increased muscle mass and fat deposition would cause an increase in PAP [18,47,48]. The data set included ultrasound values for backfat, ribeye area, rump fat, and intramuscular fat [47]. The traits were compared through a 6-trait analysis [48]. The results showed a low genetic correlation (<0.20) between PAP scores and ultrasound values for backfat, rump fat, and intramuscular fat [47]. There was a moderate, positive genetic correlation (0.25 ± 0.12) between PAP scores and ribeye area, which indicates that an increase in muscle mass and single trait selection for the ribeye area can cause an increase in PAP [47]. Pauling et al. [47] suggest that breeders recognize the unfavorable relationship between the two traits.

## 13. Future Research 

For over 100 years, HAD has been impacting the beef cattle industry [10]. In 2012, 5% of the beef cattle herd in the United States ran at high elevations [11]. With an estimated loss of USD 60 million annually [11] and a growing concern for feedlot cattle located at moderate elevations, the US beef cattle industry has been paying close attention to heart–lung function in cattle production. An increase in mean PAP scores has occurred, especially in feedlot cattle, as we put increased selection pressure on performance, and finished carcass weights have reached an all-time high [11].

There have been limited studies regarding the relationship between PAP and other traits. To date, these studies have yielded conflicting results. As the cattle industry continues to pay more attention to and learn more about PAP, it is important to have a practical application that producers can use and understand to make breeding decisions. Research to further understand the relationship between PAP and economically relevant traits would be beneficial to the beef industry. A better understanding of PAP could help decrease congestive heart failure and pulmonary hypertension in beef cattle while also decreasing economic losses. Additionally, current measurements of PAP are an invasive and expensive procedure performed by a licensed veterinarian. Alternative, less invasive procedures are needed to increase the use and application of PAP in beef production systems. 

## 14. Conclusions

High Altitude Disease is a large concern for the US beef industry, with mounting losses of up to USD 60 million annually [11]. Pulmonary arterial pressure scores can help producers select cattle that are better suited for high-elevation production systems. Furthermore, there is an increased occurrence of RHF in feedlot cattle residing at moderate elevations, which may be related to PAP and associated heart–lung function. There is very little understanding of how PAP scores are affected by other traits. Establishing a relationship between performance traits and PAP scores could prove to be very beneficial in understanding why PAP scores can be variable. Ultimately, more PAP research could help increase the knowledge surrounding PAP while also benefiting beef cattle production systems. 

## Figures and Tables

**Table 1:** Risk categories for HAD based on PAP score.

Mean PAP Score	Low Elevations (<1219 m)	Moderate Elevations (1219–1524 m)	High Elevations (1524–2286 m)	Extreme Elevations (>2286 m)
34–45 mmHg	Low Risk	Low Risk	Low Risk	Low Risk
46–49 mmHg	Moderate Risk	Moderate Risk	Moderate Risk	Moderate Risk
>50 mmHg	Moderate Risk	Moderate Risk	High Risk	High Risk

Markel, 2023 [3], Adapted from Holt and Callan, 2007 [2].

## Data Availability

Not applicable.
